# Anti-Parasitic Activities of *Allium sativum* and *Allium cepa* against *Trypanosoma b. brucei* and *Leishmania tarentolae*

**DOI:** 10.3390/medicines5020037

**Published:** 2018-04-21

**Authors:** Sonja Krstin, Mansour Sobeh, Markus Santhosh Braun, Michael Wink

**Affiliations:** Institute of Pharmacy and Molecular Biotechnology, Heidelberg University, Im Neuenheimer Feld 364, 69120 Heidelberg, Germany; sobeh@uni-heidelberg.de (M.S.); m.braun@uni-heidelberg.de (M.S.B.)

**Keywords:** garlic, onion, *Allium sativum*, *Allium cepa*, anti-parasitic activity, trypanothione, trypanothione reductase

## Abstract

**Background:** Garlics and onions have been used for the treatment of diseases caused by parasites and microbes since ancient times. Trypanosomiasis and leishmaniasis are a concern in many areas of the world, especially in poor countries. **Methods:** *Trypanosoma brucei brucei* and *Leishmania tarentolae* were used to investigate the anti-parasitic effects of dichloromethane extracts of *Allium sativum* (garlic) and *Allium cepa* (onion) bulbs. As a confirmation of known antimicrobial activities, they were studied against a selection of G-negative, G-positive bacteria and two fungi. Chemical analyses were performed using high-performance liquid chromatography (HPLC) and electrospray ionization-mass spectrometry (LC-ESI-MS/MS). **Results:** Chemical analyses confirmed the abundance of several sulfur secondary metabolites in garlic and one (zwiebelane) in the onion extract. Both extracts killed both types of parasites efficiently and inhibited the *Trypanosoma brucei* trypanothione reductase irreversibly. In addition, garlic extract decreased the mitochondrial membrane potential in trypanosomes. Garlic killed the fungi *C. albicans* and *C. parapsilosis* more effectively than the positive control. The combinations of garlic and onion with common trypanocidal and leishmanicidal drugs resulted in a synergistic or additive effect in 50% of cases. **Conclusion:** The mechanism for biological activity of garlic and onion appears to be related to the amount and the profile of sulfur-containing compounds. It is most likely that vital substances inside the parasitic cell, like trypanothione reductase, are inhibited through disulfide bond formation between SH groups of vital redox compounds and sulfur-containing secondary metabolites.

## 1. Introduction

Humans have cultivated garlics and onions since ancient times. They are well-known as food ingredients; however, because of their abundance in phytochemicals, they have also found usage in traditional folk medicine for the treatment of medical conditions like hypertension, coronary heart disease, hypercholesterolemia, cancer and infections [1,2]. Their anticancer, antioxidant, antimicrobial, antiplatelet and other biological potentials have been scientifically confirmed [3,4,5,6]. Several studies have revealed the potential of garlic (*Allium sativum*) and onion (*Allium cepa*) extracts against *Leishmania* sp. [7,8,9]. Gallwitz et al. (1999) assumed that ajoene is at least partly the source of the trypanocidal potential of *Allium sativum* [10].

**Figure 1:**
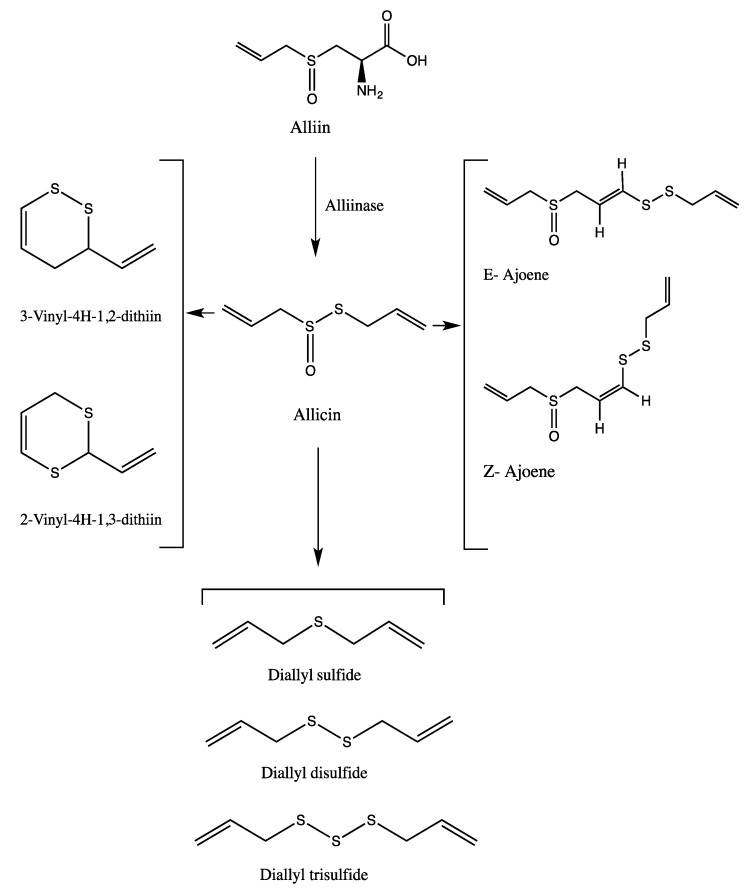
Alliinase pathway: Production of sulfur-containing secondary metabolites upon cutting garlic tissue.

**Figure 2:**
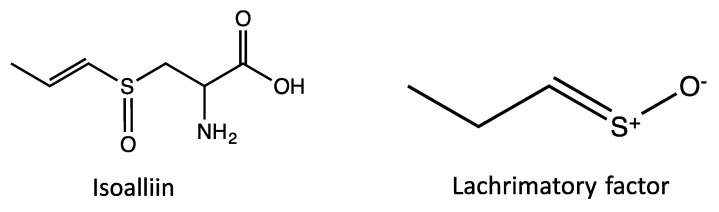

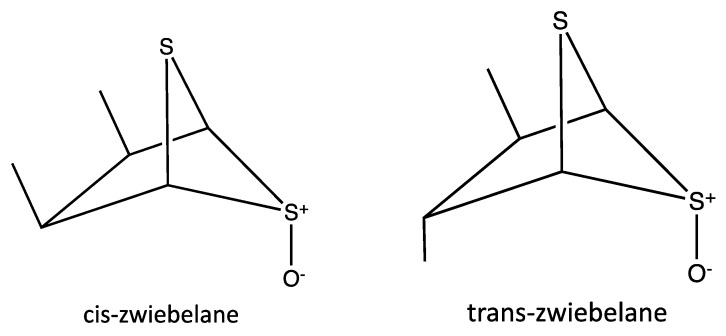
Chemical structures of sulfur-containing secondary metabolites commonly found in onion.

The odor, as well as the biological activity, of garlic and onion are attributed to their sulfur-containing secondary metabolites (SM). The major precursor of these compounds is the odorless non-protein amino acid alliin. In the intact tissue, sulfoxides like alliin and the enzyme alliinase are sequestered in different microcompartments, which are separated by thin biomembranes from the cytoplasm. Upon crushing or damaging the bulbs, the microcompartments break down, the enzyme alliinase is released and comes into contact with alliin, consequently producing volatile sulfides, which are responsible for the pungent aroma (Figure 1) [5,11,12]. Allicin and degradation products then react with each other and with intracellular thiols, generating other sulfur-containing compounds, such as derivatives and residues of cysteine [13]. In contrast, in onions the reaction starts from isoalliin. Upon cutting the tissue, the enzymatic reaction starts in analogy to garlic, producing sulfur-containing products like lachrymatory factor, *cis*-/*trans*-zwiebelanes and other thiosulfinates (Figure 2) [1,14].

Parasitic infections are a major concern, globally, especially in poor countries. *Trypanosoma brucei* is a parasite that causes, if not treated, a deadly sleeping sickness in Africa, Human African Trypanosomiasis (HAT) [15]. Leishmaniasis is a disease caused by the protozoan parasite *Leishmania*, which results in up to 30,000 deaths each year [16].

Living organisms require a reducing intracellular environment. Low molecular weight thiol-containing compounds are responsible for maintaining these conditions. Glutathione is a thiol-containing compound responsible for regulating the intracellular redox status in almost all living organisms. However, in the class Kinetoplastida, to which trypanosomes and leishmanias belong, trypanothione—an analogue of glutathione—is uniquely present and therefore serves as an interesting drug target [17,18].

In this study, we investigated the ability of dichloromethane extracts of *A. sativum* and *A. cepa* (which contain sulfur compounds) to kill trypanosomes and leishmanias. In addition, we confirmed their already-known antibacterial and antifungal activities. We additionally investigated whether the extracts could exert a synergistic or at least additive effect in combinations with common trypanocidal/leishmanicidal drugs. We provide evidence that the mode of action in parasites involves the trypanothione system.

## 2. Materials and Methods

### 2.1. Chemicals

Minimum Essential Medium (MEM), Dulbecco´s Modified Eagle´s Medium with Glutamax (DMEM), non-essential amino acids (NEAA), penicillin, streptomycin, L-glutamine and trypsin-EDTA (ethylenediaminetetraacetic acid) were purchased from Gibco^®^ Invitrogen, Darmstadt, Germany. Hemin chloride (90%) came from Merck Millipore, Darmstadt, Germany. Doxorubicin hydrochloride was acquired from the Heidelberg University Hospital. Nystatin and ampicillin were bought from AppliChem, Darmstadt, Germany. The rest of the material was obtained from Sigma-Aldrich GmbH, Steinheim, Germany.

### 2.2. Cell Lines

*Trypanosoma brucei brucei* (*T. b. brucei*) blood-stream cell line was originally obtained from Prof. Peter Overath (Max-Planck-Institut für Biologie, Tübingen, Germany). Immortalized human keratinocytes, HaCaT, were acquired in collaboration with Prof. Stefan Wölfl, Institute for Pharmacy and Molecular Biotechnology, Heidelberg, Germany. *Leishmania tarentolae*, was kindly provided by Prof. Marcel Deponte (Zentrum für Infektiologie, Parasitologie Universitätsklinikum Heidelberg, Heidelberg, Germany). In our experiments, cell lines of *Trypanosoma* and *Leishmania* were used that are not infectious for humans.

### 2.3. Standard Methods

For extract preparation, HPLC-MS/MS analyses, cell culture, MTT viability assay, antimicrobial tests and determination of *Trypanosoma brucei* trypanothione reductase (TbTR) inhibition, we followed a protocol already described in [19].

### 2.4. Reversal of Anti-Parasitic Activity

We assumed that sulfur compounds from garlic and onion could establish disulfide (-S-S-) bonds with free thiol (-SH) groups at active sites inside the parasites and therefore inhibit different vital reactions and eventually kill the parasite. Adding 2.5–250 μM of β-mercaptoethanol to the cells, newly formed disulfide bonds should be cleaved and probably reverse the cytotoxicity. MTT viability assay was carried out and the changes in the *IC*_50_ values were monitored. 

### 2.5. Mitochondrial Membrane Potential Assay

The experiment followed a protocol already described in [20,21]. Briefly, 2 × 10^6^ *T. b. brucei* cells/mL were incubated with 3, 4 and 5 μg/mL of garlic and onion extracts for 6 h. Afterwards, cells were incubated with 10 μg/mL Rh123 at 37 °C for 15 min to measure the alterations in mitochondrial membrane potential (ΔΨm). Data acquisition and analysis were performed using FACSCalibur^TM^ flow cytometer equipped with CellQuest^TM^ software. Changes in Rh123 fluorescence were quantified as percentage of fluorescence in comparison to the negative control. Negative controls were set as 100% fluorescence. Values lower than 100% correspond to depolarization of the mitochondrial membrane. CCCP (100 μM) was used as a positive control.

### 2.6. Drug Combinations

In order to detect whether the addition of garlic/onion extract to common trypanocidal (suramin, diminazene, pentamidine) and leishmanicidal (amphotericin B and pentamidine) drugs exerts a synergistic, additive or no effect at all, fixed concentrations of garlic and onion extracts were added to the serial dilutions of common trypanocidal and leishmanicidal drugs. The MTT assay was then conducted under normal conditions. Then, the combination index (*CI*) was calculated as follows:

(1) $$CI=\frac{C\left(A,X\right)}{IC\left(X,A\right)}+\frac{C\left(B,X\right)}{IC\left(X,B\right)}$$

*C* (*A*,*X*) and *C* (*B*,*X*) are the concentrations of drug A and drug B used in combination to produce a mean effect *X* (*IC*_50_). *IC* (*X*,*A*) and *IC* (*X*,*B*) are the median effect values (*IC*_50_) for single drug A and B. Combination index (*CI*) quantitatively describes synergism (*CI* < 0.90), additive effect (*CI* = 0.90–1.10), and no effect (*CI* > 1.10) [22,23].

### 2.7. Statistical Analysis

The results of experiments are shown as means ± standard deviation of at least three replicates for each measurement. Using a four-parameter logistic regression (SigmaPlot^®^ 11.0, San Jose, CA, USA), a sigmoidal curve was fitted, and the *IC*_50_, which represents 50% reduction in viability compared to non-treated cells, was calculated. Column graph data analysis was performed with Graphpad Prism 5.0 (Graphpad Software, San Diego, CA, USA). Statistical tests were performed using a Student´s *t*-test. Differences between controls and treatments were considered significant when *p*-value was smaller than 0.05.

## 3. Results

**Figure 3:**
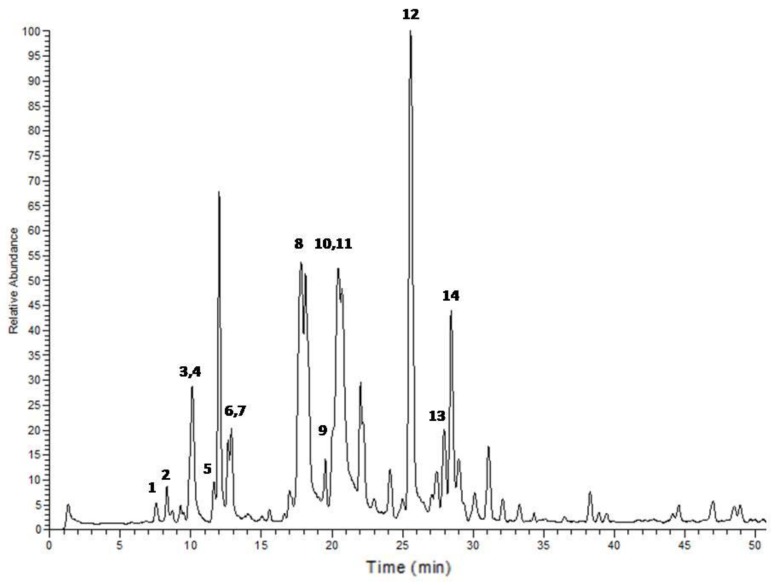
LC-ESI-MS/MS profile of the dichloromethane extract from *Allium sativum*. Peak numbers correspond to compounds listed in Table 1.

**Figure 4:**
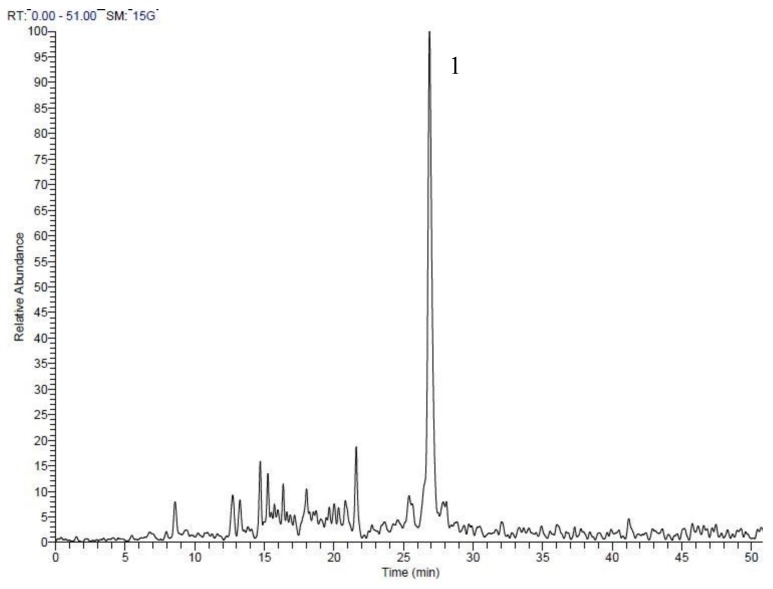
Reconstructed ion chromatogram (RIC) obtained from LC-MS in the positive ionization mode ESI (+) of the *Allium cepa* extract. Peak number corresponds to the compound in Table 2.

Chemical analysis of the *A. sativum* extract by LC-ESI-MS/MS confirmed the presence of sulfur compounds, with ajoene being the most abundant. The analysis of onion extract revealed the sulfur-containing compound zwiebelane (Table 1 and Table 2, Figure 3 and Figure 4).

**Figure 5:**
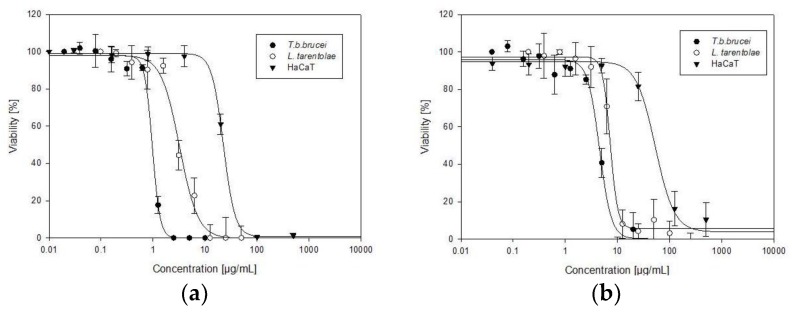
Dose-dependent trypanocidal, leishmanicidal and cytotoxic effects of (**a**) *Allium sativum*, (**b**) *Allium cepa* against *Trypanosoma brucei brucei* (*T. b. brucei*), *Leishmania tarentolae* (*L. tarentolae*) and human HaCaT cells. Data are expressed as mean of three individual experiments ± SD.

Both garlic and onion extract inhibited the growth of trypanosomes and leishmanias. We determined the trypanocidal, leishmanicidal and cytotoxic properties of the extracts using the MTT assay (Figure 5, Table 3). The garlic extract was more powerful than onion extract in all cell lines. Both extracts exerted a strong anti-parasitic activity, with *IC*_50_ values below 10 μg/mL—and that of garlic even below 1 μg/mL—and a moderate cytotoxic activity against human HaCaT cells (Table 3). The garlic extract exhibited a SI index of 23, indicating that the trypanocidal activity is more pronounced than toxicity towards human cells.

**Figure 6:**
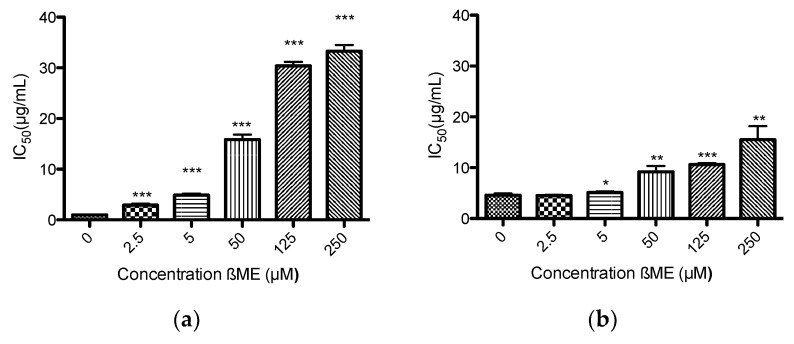
The reversal of trypanocidal effect in *Trypanosoma brucei brucei* by (**a**) garlic and (**b**) onion extracts after addition of β-mercaptoethanol. The values are expressed as mean *IC*_50_ (μg/mL) ± SD. *P* values are interpreted as: * *p* ≤ 0.05; ** *p* ≤ 0.01; *** *p* ≤ 0.001.

β-Mercaptoethanol reversed the anti-parasitic activity of both extracts in a concentration-dependent manner (Figure 6). At the highest concentration of β-mercaptoethanol (250 μM), the *IC*_50_ values of garlic and onion in *T. b. brucei* were 33.28 and 15.48 μg/mL, meaning the *IC*_50_ values were increased 35- and 3-fold, respectively.

**Figure 7:**
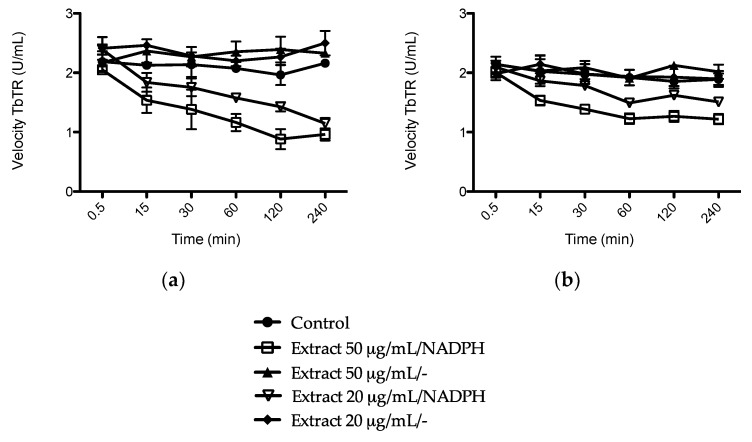
Irreversible inhibition of *Trypanosoma brucei* trypanothione reductase (TbTR) flavoenzyme by 50 and 20 μg/mL of (**a**) *Allium sativum* and (**b**) *Allium cepa* extracts. Data are shown as mean of three independent experiments ± SD.

In the inhibition assay of *Trypanosoma brucei* trypanothione reductase, garlic extract showed a substantial irreversible inhibition of the TbTR, inhibiting the activity by 55% and 47% after 4 h incubation at concentrations of 50 and 20 μg/mL, respectively. *Allium cepa* exerted a milder effect, inhibiting 35% and 20% of enzyme activity after 4 h incubation at concentrations of 50 and 20 μg/mL, respectively (Figure 7).

**Figure 8:**
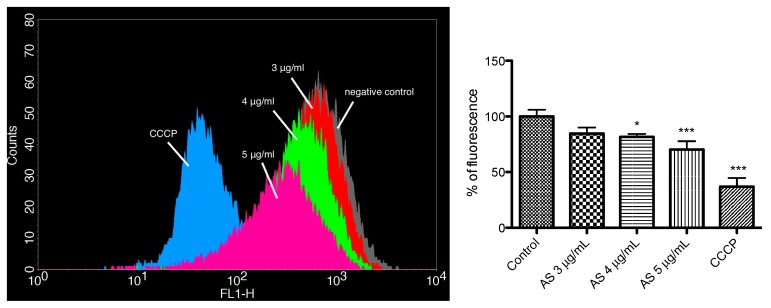
Mitochondrial membrane potential of *T. b. brucei* treated with 3, 4 and 5 μg/mL of garlic extract (AS) for 6 h and stained with Rh123. Typical histograms of three independent experiments are depicted. * *p* ≤ 0.05, significant difference relative to the control group (untreated cells). *P* values are interpreted as: * *p* ≤ 0.05; *** *p* ≤ 0.001.

The garlic extract decreased the mitochondrial membrane potential significantly in a dose-dependent manner in trypanosomes. Figure 8 shows a decrease in total Rh123 fluorescence intensity after 6 h of incubation with 3, 4, and 5 μg/mL of garlic. The onion extract failed to affect the mitochondrial membrane potential (data not shown). CCCP, which makes mitochondrial membranes leaky, was used as a positive control.

As shown in the Table 4, garlic killed the fungi *C. albicans* and *C. parapsilosis* more efficiently than the onion extract and even stronger than the positive control nystatin, with minimal inhibitory concentration (MIC) and minimal microbicidal concentration (MMC) values of 5 μg/mL. The same pattern was observed with Gram-negative bacteria, where a MIC of 40 μg/mL against *E. coli* and *P. aeruginosa* was observed. Against Gram-positive bacteria MRSA, *B. subtilis* and *S. epidermidis*, a similar activity was measured, although *Allium cepa* extract was more bactericidal for *Streptococus pyogenes* than garlic extract.

Addition of a fixed concentration of garlic to the trypanocidal drugs diminazene and pentamidine resulted in synergistic/additive effects and no effect when combined with suramin (Table 5). However, the application of 0.5 μg/mL of onion extract to a serial dilution of suramin could exert a mild synergistic effect with a CI value of 0.89. The leishmanicidal effect of amphotericin B could not be increased, no matter what extract was included in the combination. On the other hand, both extracts affected the *Leishmania tarentolae* parasites—in most cases additively—when combined with the leishmanicidal drug pentamidine.

## 4. Discussion

As expected, the phytochemical analysis of the garlic extract showed the presence of sulfur compounds, such as allicin and ajoene, to which the biological activity of garlic has been attributed [5,11,12]. On the other hand, the analysis of *Allium cepa* revealed one sulfur-containing compound, zwiebelane, which has been detected in onion extracts previously [14,28,29]. The fact that garlic produces more sulfur compounds than onion could be the explanation for the stronger activity of garlic in our study [29].

We found that garlic and onion bulb extracts have a strong anti-parasitic activity against *T. b. brucei* and *L. tarentolae*, with garlic being almost 5 times more potent against trypanosomes. We assume that the ability of these extracts to kill parasites is mediated by sulfur compounds, which are produced in the alliinase pathway after the bulb tissue was damaged. Sulfur-containing compounds can probably establish disulfide bonds (-S-S-) with free thiol groups (-SH), and thus inhibit enzymes or other proteins, which are important for survival. In trypanosomes and leishmanias, trypanothione reductase (which regulates an intracellular reducing environment) and trypanothione itself (which plays a major role in the redox system) contain thiol groups, which could be affected. Trypanothione—uniquely present in Trypanosomatidae—is responsible for detoxifying hydroperoxides and plays an important role in defense against reactive oxygen species (ROS). It contains two molecules of glutathione, connected via a molecule of spermidine. Trypanothione can be found in the parasitic cell in its disulfide (TS_2_) and dihydrotrypanothione (T[SH)z) form, but for the antioxidant activity, the reduced form is essential. Trypanothione reductase is an enzyme responsible for keeping trypanothione in its reduced form. Both trypanothione and trypanothione disulfide have a net charge of +1, while glutathione (GSH) and glutathione disulfide (GSSG) have a net charge of −2, which is probably the reason for the high specificity of the two enzymes [17,30]. We already showed in our previous study that dichloromethane extracts from *Allium ursinum* and *Tulbaghia violacea* are capable of inhibiting trypanothione reductase and consequently mediate a growth inhibition of the parasites [19]. By adding β-mercaptoethanol, which can reduce disulfide bonds, we managed to reverse the cytotoxic effect. We postulate that β-mercaptoethanol can split newly formed disulfide bonds between trypanothione (and/or trypanothione reductase) and sulfur compounds from the extracts; trypanothione becomes consequently active again, leading to a higher survival of the parasite. To further corroborate our hypothesis, we show that the activity of trypanothione reductase is irreversibly reduced in the presence of garlic, while only moderately in the presence of onion extract. The results confirm our hypothesis that the sulfur-containing compounds produced in the alliinase pathway are responsible for the anti-parasitic activity.

Concerning the antimicrobial activity, *Allium sativum* was more active than *Allium cepa*, which agrees with the literature [31]. In our study we could confirm the known antibacterial and antifungal activity of both extracts [32,33].

Furthermore, we evaluated the cytotoxic activity of both extracts against human keratinocytes, to determine whether these extracts have a potential to be used therapeutically as topical agents for skin infections. Onion extract exerted a milder cytotoxicity; however, the selectivity index is more favorable for the garlic extract, meaning that garlic extract would probably have less side effects.

Garlic extract decreased the mitochondrial membrane potential in trypanosomes. This result could indicate that apoptosis-like processes are also triggered by garlic, based on the fact that the decrease could be initiating apoptosis, or could be one of the consequences of the apoptosis [34]. This process was also demonstrated in protozoa, and not only in metazoa [35]. Our combination experiments of the plant extracts with established therapeutics shows that 50% of the tested combinations resulted in a synergistic/additive effect. This means that garlic and onion could potentially be used in combination therapies with common trypanocidal/leishmanicidal drugs in order to enhance their anti-parasitic activity.

## 5. Conclusions

In conclusion, our results confirmed that garlic can kill bacteria and fungi. Both extracts showed a potent trypanocidal and leishmanicidal activity. The activity is most likely mediated via an inhibition of vital redox compounds such as trypanothione and/or trypanothione reductase inside the parasites. We assume that disulfide bonds are formed between the thiols of garlic and onions and trypanothione and TR, consequently decreasing the level of free thiol groups and inhibiting the redox system, thereby leading to the death of parasites. Further studies using multivariate methods relating the activity results and the spectroscopic data can aid to elucidate which compounds from extracts are responsible for the activities. The promising synergistic activities of garlics and onions with trypanocidal/leishmanicidal drugs need to be corroborated in animal experiments. If confirmed, they might be relevant in a therapeutic context.

## Figures and Tables

**Table 1:** Identification of sulfur-containing compounds in *Allium sativum* extract by LC-ESI-MS/MS.

Peak No.	t_R_	Area %	[M + H]^+^	Tentative identification	Reference
1	7.54	0.69	227	1-Ethyl-2-(3-(propylsulfinyl)propyl)disulfane	Tentative
2	8.31	1.12	249	1-Allyl-2-((1E,3E)-4-(vinyldisulfanyl)buta-1,3-dien-1-yl)disulfane	Tentative
3	9.89	0.64	137	Methanesulfinothioic acid S-(E)-1-propenyl ester	[24]
4	10.08	6.34	137	Methanesulfinothioic acid S-(Z)-1-propenyl ester	[24]
5	11.68	1.29	137	S-methyl 1-propenesulfinothioate/S-1-propenyl methanesulfinothioate	[24]
6	12.64	2.08	251	Gamma-l-glutamyl-l-cysteine	[25]
7	12.92	3.06	251	Gamma-l-glutamyl-l-cysteine	[25]
8	17.88	23.66	163	Allicin	[25]
9	20.12	1.25	209	(E)-1-allyl-2-(3-(methylesulfinyl)prop-1-en-1-yl)disulfane	Tentative
10	20.37	11.78	163	2-Propene-1-sulfinothioic acid S-(E)-1-propenyl ester	[24]
11	20.53	12.89	163	Propene-1-sulfinothioic acid S-(Z)-1-propenyl ester	[24]
12	25.59	23.39	235	Ajoene	[26]
13	27.95	3.31	237	(E)-1-Propenyl 1-(1-propenylsulfinyl)propyl disulfide	[27]
14	28.45	8.49	237	2-Propenyl 1-(2-propenylsulfinyl) propyl disulfide	[27]

**Table 2:** Identification of sulfur-containing compounds in *Allium cepa* extract by LC-ESI-MS/MS.

Peak No.	t_R_	[M + H]^+^	Proposed Compound	Reference
1	26.87	163	(*cis/trans*)-zwiebelane	[28]

**Table 3:** Trypanocidal, leishmanicidal and cytotoxic activity of *Allium sativum* and *Allium cepa* extracts against *Trypanosoma brucei brucei* (*T. b. b.*), *Leishmania tarentolae* (*L. t.*) and HaCaT cells. The values are expressed as mean *IC*_50_ (μg/mL) ± SD; NT: not tested.

Sample	*IC*_50_ *T. b. b.*	*IC*_50_ *L. t.*	*IC*_50_ HaCaT	Selectivity Index	
				HaCaT/*T. b. b.*	HaCaT/*L. t.*
*Allium sativum*	0.95 ± 0.04	2.89 ± 0.4	22.27 ± 1.61	23	8
*Allium cepa*	4.59 ± 0.34	7.23 ± 0.78	44.56 ± 3.06	10	6
Suramin	0.13 ± 0.01	NT	NT	/	/
Amphotericin B	NT	0.13 ± 0.02	NT	/	/
Doxorubicin	NT	NT	1.04 ± 0.35	/	/

**Table 4:** Antimicrobial activity of *Allium sativum* and *Allium cepa* extracts against different G-positive, G-negative bacteria and *Candida* yeasts in microdilution assays. Data are given in μg/mL of minimal inhibitory concentration (MIC) and minimal microbicidal concentration (MMC) values. Positive controls were ciprofloxacin, ampicillin and nystatin; NT: not tested.

Gram Type	Sample	*A. sativum*		*A. cepa*		Ciprofloxacin	Ampicillin	Nystatin
	Indicator Strain							
		MIC	MMC	MIC	MMC	MIC	MIC	MIC
+	*Bacillus subtilis*	40	160	40	>320	≤0.03	≤0.03	NT
+	MRSA	40	>320	320	>320	0.03	16	NT
+	MRSA CI	80	>320	160	>320	4	16	NT
+	*Staphylococcus epidermidis*	40	>320	80	>320	0.03	0.5	NT
+	*Enterococcus faecalis*	160	>320	>320	>320	0.5	1	NT
+	VRE	320	>320	>320	>320	0.5	1	NT
+	*Streptococcus pyogenes*	80	160	40	40	0.13	<0.03	NT
-	*Escherichia coli*	40	160	>320	>320	≤0.03	4	NT
-	*Escherichia coli* EHEC	40	>320	>320	>320	≤0.03	4	NT
-	*Klebsiella pneumoniae*	80	>320	>320	>320	0.125	>64	NT
-	*Klebsiella pneumoniae* CI	80	>320	>320	>320	<0.03	32	NT
-	*Pseudomonas aeruginosa*	40	>320	160	>320	≤0.03	>64	NT
F	*Candida albicans*	5	5	160	160	NT	NT	10
F	*Candida parapsilosis*	5	5	160	160	NT	NT	10

**Table 5:** Combinations of *Allium sativum* (garlic) and *Allium cepa* (onion) extracts with common trypanocidal (diminazene, pentamidine and suramin) and leishmanicidal (amphotericin B and pentamidine) drugs.

Extract	*IC*_50_ ± SD Extract Alone (μg/mL)	Drug	*IC*_50_ ± SD Drug Alone (μM)	Fixed Concentration of the Extract (μg/mL)	*IC*_50_ ± SD Drug in Combination (μM)	CI Value at *IC*_50_	Interpretation
	*T. b. brucei*						
*Allium cepa*	4.59 ± 0.34	Diminazene	0.24 ± 0.01	0.5	0.19 ± 0.09	0.90	Additive
				1	0.24 ± 0.04	1.22	No effect
		Pentamidine	0.07 ± 0.01	0.5	0.11 ± 0.01	1.68	No effect
				1	0.09 ± 0.01	1.50	No effect
		Suramin	0.09 ± 0.01	0.5	0.07 ± 0.01	0.89	Synergism
				1	0.07 ± 0.01	1.00	Additive
	*L. tarentolae*						
	7.23 ± 0.78	Amphotericin B	0.14 ± 0.02	0.75	0.15 ± 0.02	1.17	No effect
				1.5	0.15 ± 0.02	1.28	No effect
		Pentamidine	4.01 ± 0.86	0.75	3.82 ± 0.81	1.06	Additive
				1.5	3.71 ± 0.94	1.13	No effect
	*T. b. brucei*						
*Allium sativum*	0.95 ± 0.04	Diminazene	0.24 ± 0.01	0.1	0.20 ± 0.05	0.94	Additive
				0.2	0.19 ± 0.03	1.00	Additive
		Pentamidine	0.07 ± 0.01	0.1	0.05 ± 0.02	0.82	Synergism
				0.2	0.05 ± 0.02	0.92	Additive
		Suramin	0.09 ± 0.01	0.1	0.1 ± 0.01	1.22	No effect
				0.2	0.1 ± 0.01	1.32	No effect
	*L. tarentolae*						
	2.89 ± 0.4	Amphotericin B	0.14 ± 0.02	0.32	0.14 ± 0.02	1.11	No effect
				0.64	0.13 ± 0.01	1.15	No effect
		Pentamidine	4.01 ± 0.86	0.32	3.5 ± 0.45	0.98	Additive
				0.64	3.18 ± 0.46	1.01	Additive
